# An explainable predictive machine learning model of osteopenia for perimenopausal women based on clinical data: a retrospective single-center study

**DOI:** 10.3389/fendo.2026.1817729

**Published:** 2026-05-29

**Authors:** Xiaoling Zhuo, Huixian Zeng, Huoqiang Chen, Yunlin Wang, Zhenhua Feng, Quanhui Liang

**Affiliations:** 1 Department of Clinical Laboratory, The First People’s Hospital of Foshan (Foshan Hospital Affiliated to Southern University of Science and Technology), School of Medicine, Southern University of Science and Technology, Foshan, Guangdong, China; 2 Foshan Key Laboratory of Skin Tissue Engineering and Precision Diagnosis and Treatment of Infectious Skin Diseases, Foshan, China; 3 Department of Endocrinology, The Second People’s Hospital of Foshan, Affiliated Foshan Hospital of Guangdong Pharmaceutical University, Foshan, Guangdong, China

**Keywords:** blood biochemical indicators, low bone density, machine learning, osteoporosis, prediction model

## Abstract

**Background:**

Osteoporosis is increasingly prevalent, yet early detection remains difficult. This study aimed to develop a machine learning (ML)-based model for identifying individuals at risk of osteopenia using clinical data.

**Methods:**

Female participants who were aged 45 years and above with complete femoral neck BMD data were included. A total of 1,108 participants were divided into training (70%) and test (30%) datasets. Various ML-based algorithms (random forest (RF), least absolute shrinkage and selection operator (LASSO), gradient boosting decision tree (GBDT) were used to identify key predictors of osteopenia, including clinical and biochemical markers. Model performance was evaluated based on area under the curve (AUC), accuracy, sensitivity, specificity, precision, and F1 score, with SHAP analysis used for feature importance.

**Results:**

Totally, 17 predictors were identified, with RF demonstrating the best performance (AUC: 0.978 in training, 0.933 in validation datasets). Key predictors of osteopenia included menopause, age, procollagen type I N-terminal propeptide (PINP), beta-crosslaps (β-CTX), height, and estimated glomerular filtration rate (eGFR). RF outperformed other models in both identify accuracy and clinical utility, with an AUC of 0.90 and AUPR of 0.93 for the top six features. A web-based calculator was developed for clinical use.

**Conclusion:**

The RF model can effectively identify osteopenia and could improve early detection of osteoporosis. This model, with its integration into clinical practice, has the potential to enhance patient outcomes and reduce osteoporosis-related risks.

## Introduction

Osteoporosis, a systemic metabolic bone disorder, is characterized by a progressive decline in bone mineral density (BMD) and deterioration of bone microarchitecture, leading to fragile bones that are highly susceptible to fractures. These fractures, particularly in the spine, hip, and wrist, can severely impair an individual’s functional ability and quality of life, with significant long-term consequences including disability and increased mortality risk (1). Osteoporosis is often considered a silent disease, with many individuals unaware of their condition until a fracture occurs, underscoring the importance of early detection and proactive management (2).

Globally, osteoporosis and osteopenia affect a substantial portion of the population, particularly postmenopausal women. A large-scale study involving 260,810 participants from 30 countries found that the prevalence of osteopenia was 40.4%, with women exhibiting higher rates (39.4%) compared to men (3). In China, osteopenia prevalence is notably high, with a survey in Hubei Province revealing that 65.8% of women aged 50 and older had osteopenia or osteoporosis, far surpassing the rates seen in men (44.96%) (3). These figures reflect a growing concern as the aging population in China and other countries continues to increase, contributing to a rising prevalence of osteoporosis, which places immense social and economic burdens on healthcare systems worldwide.

Beyond its role in increasing fracture risk, low BMD, whether osteopenia or osteoporosis, is also linked to various systemic diseases, including cardiovascular disease (4), liver cirrhosis (5), chronic kidney disease (6), and neurodegenerative disorders (7). Given the broad implications of low bone health, the timely identification and management of osteopenia are crucial not only to prevent fractures but also to reduce the risks associated with these comorbidities.

Despite the well-established importance of early osteoporosis detection, existing screening methods remain limited in terms of accessibility, affordability, and accuracy. Commonly used instruments such as the Osteoporosis Risk Assessment Instrument (ORAI), the Osteoporosis Self-assessment Tool (OST), and the Fracture Risk Assessment Tool (FRAX) rely on a limited range of clinical risk factors like age and body weight, which limits their ability to accurately identify osteoporosis in diverse populations. While these tools can help identify high-risk individuals, they are often criticized for their lack of sensitivity and specificity, especially when applied to specific groups, such as perimenopausal women (8).

Currently, dual-energy X-ray absorptiometry (DXA) is considered the gold standard for diagnosing osteoporosis by measuring BMD. However, DXA has significant limitations, including high costs, the need for specialized equipment and skilled personnel, space requirements, and radiation exposure. Moreover, DXA machines are not evenly distributed worldwide, and in regions like China, there is less than one DXA machine per million people, making widespread screening impractical (9). This scarcity is even more pronounced in rural and underserved areas, where access to healthcare services is limited. Consequently, these logistical challenges highlight the urgent need for alternative, cost-effective methods to assess osteoporosis risk that can be deployed at the community level, particularly in primary healthcare settings.

To address the limitations of traditional screening methods, this study aims to develop and validate a machine learning (ML)-based model for identifying osteopenia risk, specifically targeting perimenopausal women. ML, which mimics human cognition by identifying patterns and learning from data, has shown considerable promise in clinical applications, particularly in predicting disease outcomes. ML models can process large datasets, incorporate complex variables, and improve prediction accuracy compared to traditional tools. However, a key challenge with ML models is their “black-box” nature, where the decision-making process is not easily interpretable (8). To overcome this limitation, we employed the Shapley Additive exPlanation (SHAP) method, which provides transparency by explaining the contributions of individual variables, thus enhancing the interpretability of the model.

By developing an accessible ML-based tool, we aim to provide healthcare professionals, particularly those in grassroots healthcare settings, with a practical method to identify osteopenia risk. This tool could support clinicians in making more informed decisions about when to screen for osteoporosis, what interventions are necessary, and how to manage patients with early signs of bone loss. Moreover, the model could reduce unnecessary referrals for costly DXA scans, alleviating some of the burdens on healthcare systems, particularly in resource-limited settings.

The primary objective of this study was to develop a robust and validated machine learning-based model for identifying osteopenia in perimenopausal women aged 45 and above. The model incorporates key clinical and laboratory variables, including age, gender, body mass index (BMI), and biomarkers, to forecast osteopenia status. The study aimed to provide a tool that not only enhances early diagnosis but also empowers healthcare providers to implement timely interventions and improve patient outcomes. Ultimately, the adoption of this tool in clinical practice could reduce the incidence of osteoporotic fractures, lower healthcare costs, and improve the quality of life for millions of individuals at risk.

## Materials and methods

### Study design and population

**Figure 1:**
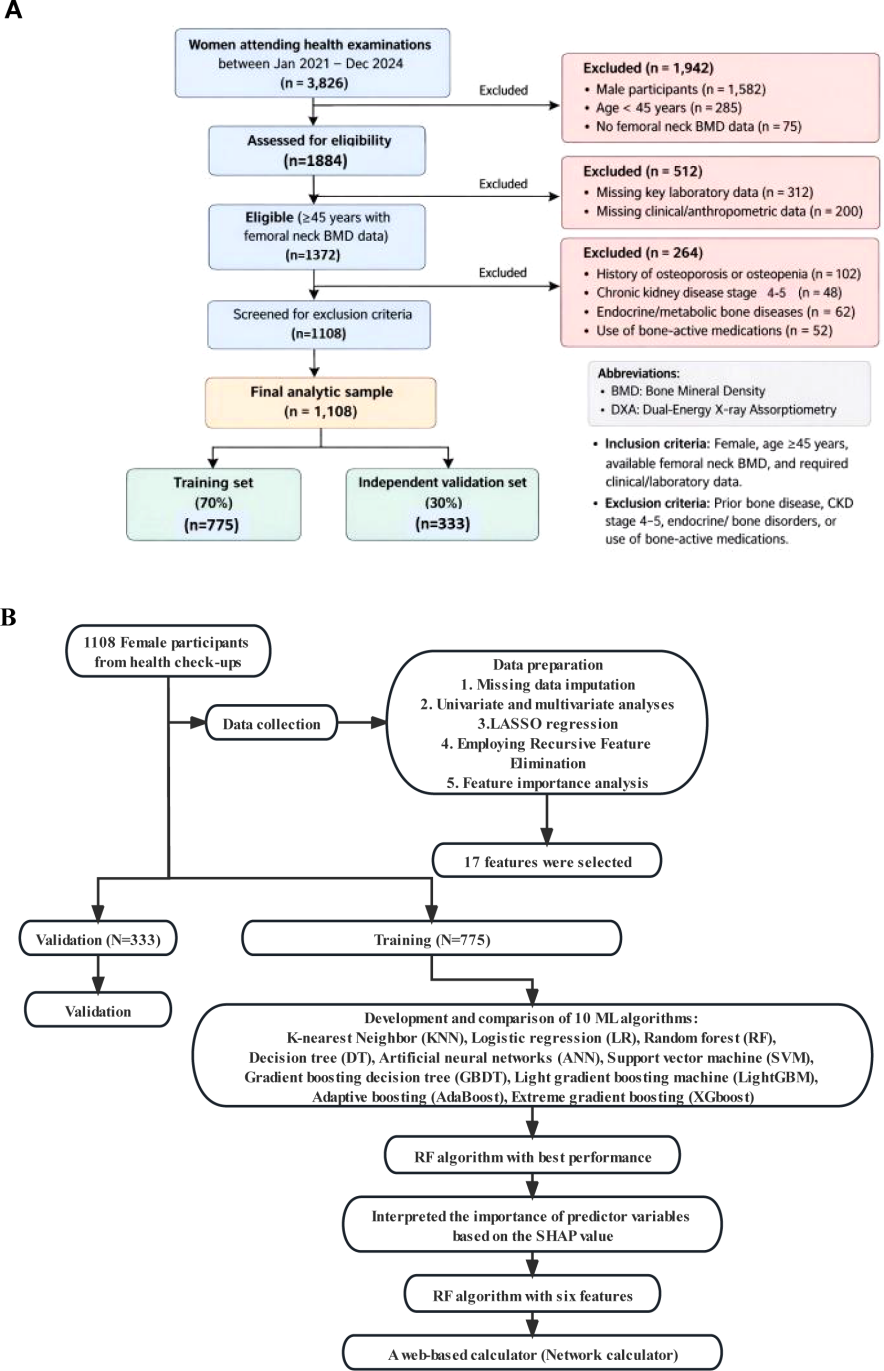
**(A)** Flowchart of participant selection. **(B)** Overview of the study design and analytical workflow.

This retrospective cohort study consecutively enrolled female participants over the age of 45 years from the health management center of the First People’s Hospital of Foshan, a prominent tertiary medical institution catering to both urban and rural populations, over a span extending from January 2021 to December 2024. Menopausal status was defined as the absence of menstruation for at least 12 consecutive months, based on clinical history recorded at the time of examination. The investigation concentrated exclusively on obtaining and analyzing comprehensive bone mineral density (BMD) measurements for the lumbar spine, femoral neck, and total hip. Stringent inclusion and exclusion criteria were precisely applied to ensure the homogeneity and relevance of the study population. The following inclusion criteria were used (1): female patients aged 45 years and above (2); complete femoral neck BMD measurements obtained by DXA (3); the presence of corresponding clinical and laboratory data needed to be analyzed. Only individuals who were treated with osteoporosis pharmacotherapies (bisphosphonates, teriparatide, hormone replacement therapy (HRT), selective estrogen receptor modulators (SERMs), or corticosteroids were excluded, as well as those with conditions (anorexia nervosa, Cushing syndrome, hyperparathyroidism, or chronic kidney disease)—**Figure 1A**. Additionally, individuals with secondary causes of osteoporosis, such as osteogenesis imperfecta, systemic rheumatism, hematological malignancies, including multiple myeloma, leukemia, and lymphoma, as well as conditions, such as systemic mast cell hyperplasia, prior documented osteoporotic fractures, gastrointestinal or biliary disorders, or those with adverse reactions to specific medications (including anticonvulsants), were also excluded. Women with diabetes mellitus were not excluded from the study. The comprehensive survey design, encompassing all methodological steps and variables, is clearly illustrated in **Figure 1B**. The study protocol received approval from the Ethical Committee, in compliance with the Declaration of Helsinki (Ethical Approval No. 2026P051).

### Data collection and feature selection

The study involved a comprehensive array of 55 admission parameters, which were systematically classified into three primary categories (1): Basic characteristics, which comprised demographic and anthropometric data, comprising age, age at menopause, and body mass index (BMI) (2); Admission Laboratory Data, which spanned a broad spectrum of biochemical and physiological markers, such as bone turnover markers, including Serum N-terminal propeptides of type I collagen (PINP) and β-collagen special sequence (β-CTX), along with pivotal biochemical indices such as total 25-hydroxyvitamin D, parathyroid hormone (PTH), routine blood parameters, liver function markers (e.g., alanine aminotransferase (ALT), aspartate aminotransferase (AST), total bilirubin (TB), direct bilirubin (DB), gamma-glutamyl transpeptidase (GGT), and alkaline phosphatase (ALP)), renal function indicators (urea nitrogen (UREA), creatinine (CREA), and cystatin C), electrolytes (potassium, sodium, chloride, calcium, phosphorus), lipids (total cholesterol (TC), triglycerides (TG), high-density lipoprotein (HDL), low-density lipoprotein (LDL)), muscle enzymes (creatine kinase (CK), lactate dehydrogenase (LDH)), proteins (total protein (TP), albumin, globulin), and metabolic parameters including glucose and uric acid (UA); and (3) Composite Indices, which were derived from a synthesis of the aforementioned parameters, including advanced metrics such as the estimated glomerular filtration rate (eGFR) (10), the triglyceride glucose index (TyG), Systemic Immune-Inflammation Index (SII), Systemic Inflammation Response Index (SIRI), Uric Acid to High-Density Lipoprotein Cholesterol Ratio (UHR), and the Plasma Arterial Inflammation Index (AIP), thereby providing a multi-dimensional assessment of the subjects’ physiological and metabolic status. The intra-assay and inter-assay coefficients of variation for PINP and β-CTX were within acceptable limits (<5% and <8%, respectively), according to the manufacturer’s specifications. Reference ranges for these markers were based on healthy premenopausal women provided by the assay manufacturer.

All serum biomarkers were measured from blood samples collected after an overnight fast (at least 8 hours) between 8 a.m. and 11 a.m. to minimize the impact of diurnal variation. All continuous variables, including clinical and laboratory parameters, were standardized using z-score normalization. Variables with a missing rate greater than 30% were excluded from the analysis. After this exclusion step, a total of 48 variables remained and were used for subsequent feature selection and model development. It should be noted that **Table 1** presents a broader set of baseline variables prior to exclusion for descriptive comparison purposes. The proportion of missing data for each variable is provided in **Supplementary Table 1**. All continuous variables were standardized using z-score normalization before model development. Only participants with complete data for the selected variables were included in the final analysis.

**Table 1:** Comparison of demographic characteristics and clinical characteristics between the osteopenia group and the normal group, and between the training and test sets.

**Characteristic**	**Overall, N = 1108**	**Normal group** **(n=500)**	**Osteopenia group (n=608)**	**P-value**	**Training set,** **(n=775)**	**Test set,** **(n=333)**	**P-value**
Age, Median (IQR)	52 (48, 58)	48 (46, 51)	56 (52, 62)	< 0.001	52 (48, 58)	52 (48, 58)	0.807
Menopause, n (%)				< 0.001			0.367
M0	353 (32)	346 (69)	7 (1)		113 (34)	240 (31)	
M1	755 (68)	154 (31)	601 (99)		220 (66)	535 (69)	
BMI, Median (IQR)	22.7 (21.03, 24.86)	23.05 (21.33, 24.97)	22.45 (20.83, 24.71)	0.006	22.51 (20.97, 24.62)	22.77 (21.09, 24.89)	0.341
GLU, Median (IQR)	5.07 (4.78, 5.43)	5.02 (4.74, 5.32)	5.11 (4.8, 5.51)	< 0.001	5.05 (4.76, 5.44)	5.07 (4.79, 5.43)	0.689
ADPN, Median (IQR)	9.36 (7.39, 11.02)	9.46 (7.58, 11.56)	9.23 (7.25, 10.68)	0.008	9.36 (7.4, 10.84)	9.34 (7.38, 11.16)	0.684
INS, Median (IQR)	8.84 (6.57, 12.35)	8.95 (6.98, 12.21)	8.66 (6.19, 12.49)	0.151	8.73 (6.73, 11.86)	8.91 (6.52, 12.56)	0.343
FT4, Median (IQR)	15.71 (15.09, 17.13)	15.48 (15.03, 16.27)	15.98 (15.2, 17.49)	< 0.001	15.72 (15.09, 17.27)	15.71 (15.12, 17.09)	0.985
FT3, Median (IQR)	4.89 (4.65, 5.05)	4.92 (4.76, 5.05)	4.86 (4.59, 5.06)	0.003	4.89 (4.62, 5.07)	4.9 (4.67, 5.04)	0.704
TSH, Median (IQR)	2.05 (1.46, 2.56)	2.11 (1.6, 2.53)	2.0 (1.39, 2.59)	0.11	2.03 (1.44, 2.52)	2.06 (1.49, 2.59)	0.332
TPINP, Median (IQR)	51.72 (38.33, 68.72)	44.47 (33.61, 56.97)	59.55 (43.73, 75.35)	< 0.001	51.64 (38.08, 70.24)	51.8 (38.36, 67.49)	0.749
β-CTX, Median (IQR)	0.39 (0.26, 0.54)	0.32 (0.23, 0.45)	0.44 (0.31, 0.6)	< 0.001	0.38 (0.26, 0.54)	0.39 (0.26, 0.54)	0.852
iPTH, Median (IQR)	26.26 (17.4, 29.79)	27.53 (21.37, 29.93)	23.91 (15.8, 29.48)	< 0.001	26.43 (17.1, 29.79)	26.19 (17.48, 29.77)	0.979
VITD, Median (IQR)	26.76 (21.49, 33.2)	25.39 (20.7, 31.21)	27.76 (22.74, 34.12)	< 0.001	27.31 (22, 33.23)	26.52 (21.44, 33.09)	0.484
TP, Median (IQR)	73.1 (70.7, 75.8)	72.9 (70.8, 75.9)	73.1 (70.6, 75.73)	0.998	72.6 (70, 75.3)	73.3 (70.8, 76)	0.018
ALB, Median (IQR)	43.9 (42.5, 44.9)	43.85 (42.5, 44.8)	43.95 (42.48, 45.1)	0.281	43.8 (42.3, 44.8)	43.9 (42.6, 45)	0.126
TBIL, Median (IQR)	12 (10, 14.7)	11.9 (9.6, 14.83)	12.2 (10.2, 14.62)	0.251	11.9 (10, 14.9)	12 (10, 14.7)	0.995
ALT, Median (IQR)	17 (13, 22)	16 (13, 21)	18 (14, 24)	< 0.001	16 (13, 22)	17 (13, 22)	0.846
AST, Median (IQR)	20 (17, 24)	19 (17, 22.25)	21 (18, 25)	< 0.001	20 (17, 24)	20 (17, 24)	0.329
GGT, Median (IQR)	19 (14, 25)	18 (13, 23)	19 (15, 27)	< 0.001	19 (14, 25)	18 (14, 25)	0.254
ALP, Median (IQR)	66 (57, 77.12)	60.6 (54.2, 69)	72.65 (61, 83)	< 0.001	66 (57, 78)	66 (57, 77)	0.786
TG, Median (IQR)	1.09 (0.81, 1.49)	1.04 (0.76, 1.39)	1.16 (0.84, 1.61)	< 0.001	1.11 (0.81, 1.44)	1.09 (0.8, 1.52)	0.633
CHOL, Median (IQR)	5.24 (4.66, 5.92)	5.08 (4.53, 5.64)	5.43 (4.77, 6.16)	< 0.001	5.22 (4.54, 6)	5.25 (4.7, 5.89)	0.642
HDLC, Median (IQR)	1.43 (1.21, 1.65)	1.43 (1.22, 1.66)	1.42 (1.2, 1.65)	0.844	1.44 (1.2, 1.63)	1.42 (1.22, 1.66)	0.956
LDLC, Median (IQR)	3.32 (2.71, 3.98)	3.22 (2.65, 3.83)	3.41 (2.74, 4.06)	0.001	3.33 (2.65, 3.92)	3.32 (2.73, 4)	0.626
CK, Median (IQR)	77.2 (67.68, 93.12)	73.9 (65.97, 88.35)	81.95 (69.3, 101)	< 0.001	79 (66, 95)	77.1 (68, 92.65)	0.821
LDH, Median (IQR)	162.2 (153.9, 173)	162.25 (154.57, 170.12)	162.15 (152, 176)	0.474	162.7 (153.4, 171.7)	162 (153.95, 173)	0.972
CRP, Median (IQR)	1.07 (0.4, 2.07)	1.17 (0.46, 2.08)	0.98 (0.33, 2.04)	0.124	1.11 (0.45, 2.1)	1.06 (0.38, 2.06)	0.496
FER, Median (IQR)	110.9 (55.1, 164.52)	56.95 (39.88, 102.83)	145.25 (105.27, 188.17)	< 0.001	109.6 (51.5, 161.7)	111 (57.6, 166.25)	0.327
FE, Median (IQR)	17.88 (14.43, 20.72)	18.05 (14.16, 20.97)	17.74 (14.62, 20.57)	0.83	18.23 (15.01, 21.07)	17.66 (14.34, 20.59)	0.057
UIBC, Median (IQR)	46.52 (38.01, 49.18)	47.45 (43.89, 49.51)	45.14 (35.41, 48.71)	< 0.001	46.34 (38.01, 49.23)	46.58 (38.08, 49.14)	0.751
eGFR, Median (IQR)	102.55 (95.28, 107.23)	105.74 (100.79, 110.22)	99.7 (93.32, 104.36)	< 0.001	102 (94.83, 107.95)	102.55 (95.97, 107.09)	0.924
UREA, Median (IQR)	5.18 (4.44, 6.07)	4.94 (4.18, 5.8)	5.42 (4.63, 6.31)	< 0.001	5.23 (4.56, 5.98)	5.15 (4.38, 6.12)	0.701
UA, Median (IQR)	310 (271, 360)	307.5 (269, 356.25)	311 (274.75, 361.25)	0.35	315 (273, 369)	308 (269.5, 355.5)	0.296
CYSC, Median (IQR)	0.83 (0.76, 0.91)	0.78 (0.74, 0.85)	0.86 (0.8, 0.97)	< 0.001	0.83 (0.76, 0.92)	0.82 (0.76, 0.91)	0.796
CA, Median (IQR)	2.34 (2.27, 2.37)	2.34 (2.27, 2.37)	2.33 (2.26, 2.37)	0.925	2.34 (2.27, 2.37)	2.34 (2.27, 2.37)	0.212
IP, Median (IQR)	1.22 (1.16, 1.27)	1.23 (1.18, 1.26)	1.22 (1.13, 1.29)	0.656	1.22 (1.15, 1.27)	1.23 (1.16, 1.28)	0.307
K, Median (IQR)	4.38 (4.3, 4.48)	4.37 (4.31, 4.44)	4.38 (4.27, 4.52)	0.309	4.38 (4.3, 4.5)	4.37 (4.3, 4.47)	0.289
NA, Median (IQR)	142.6 (141.4, 143.5)	142.2 (141.2, 143)	142.9 (141.6, 144.12)	< 0.001	142.6 (141.3, 143.5)	142.6 (141.4, 143.55)	0.641
CL, Median (IQR)	106.7 (105.9, 107.5)	106.6 (106.1, 107.1)	106.8 (105.65, 108.1)	0.01	106.7 (105.8, 107.3)	106.7 (105.9, 107.5)	0.449
ESR, Median (IQR)	15.15 (11, 20)	14.25 (10, 19)	16 (12, 20.45)	< 0.001	15 (11, 19.5)	15.2 (11, 20)	0.627
WBC, Median (IQR)	5.46 (4.73, 6.59)	5.64 (4.87, 6.83)	5.36 (4.65, 6.42)	0.002	5.56 (4.87, 6.65)	5.39 (4.66, 6.53)	0.144
RBC, Median (IQR)	4.51 (4.28, 4.76)	4.47 (4.24, 4.74)	4.52 (4.32, 4.79)	0.008	4.52 (4.3, 4.79)	4.51 (4.28, 4.76)	0.758
HGB, Median (IQR)	131 (124, 137.25)	130 (122, 136.25)	132.8 (126, 138)	< 0.001	132 (124, 138)	131 (124, 137)	0.765
HCT, Median (IQR)	0.4 (0.38, 0.42)	0.4 (0.38, 0.42)	0.41 (0.39, 0.42)	< 0.001	0.4 (0.38, 0.42)	0.4 (0.38, 0.42)	0.675
PLT, Median (IQR)	251 (218, 288)	260 (228, 301)	243.85 (211, 278)	< 0.001	253 (222, 288)	250 (216, 287)	0.341
NEU, Median (IQR)	3.13 (2.56, 3.95)	3.29 (2.69, 4.19)	3.01 (2.49, 3.76)	< 0.001	3.22 (2.6, 4.12)	3.09 (2.54, 3.91)	0.104
LYM, Median (IQR)	1.81 (1.53, 2.14)	1.77 (1.5, 2.13)	1.82 (1.56, 2.15)	0.059	1.79 (1.54, 2.13)	1.81 (1.52, 2.15)	0.717
MONO, Median (IQR)	0.31 (0.25, 0.38)	0.31 (0.26, 0.39)	0.31 (0.24, 0.38)	0.014	0.31 (0.27, 0.39)	0.31 (0.25, 0.38)	0.139
TyG, Median (IQR)	8.41 (8.07, 8.76)	8.32 (8.01, 8.66)	8.47 (8.12, 8.84)	< 0.001	8.41 (8.06, 8.74)	8.41 (8.07, 8.78)	0.727
SII, Median (IQR)	435.34 (319.66, 602.54)	493.01 (373.8, 671.88)	404.09 (293.27, 544.21)	< 0.001	455 (325.69, 607.16)	423.5 (312.17, 599.09)	0.11
SIRI, Median (IQR)	0.53 (0.38, 0.79)	0.58 (0.43, 0.88)	0.49 (0.36, 0.71)	< 0.001	0.56 (0.42, 0.84)	0.51 (0.37, 0.77)	0.015
UHR, Median (IQR)	216.89 (171.29, 277.33)	212.83 (172.17, 274.66)	220.46 (171.06, 279.65)	0.754	225.44 (173.53, 278.29)	214.36 (170.96, 276.83)	0.378
AIP, Median (IQR)	-0.11 (-0.29, 0.06)	-0.14 (-0.31, 0.04)	-0.09 (-0.27, 0.09)	0.006	-0.11 (-0.28, 0.03)	-0.12 (-0.3, 0.07)	0.827

Prior to LASSO regression, a multi-step variable preselection process was conducted. First, variables with more than 30% missing data were excluded, resulting in 48 variables. Second, univariate regression analysis was performed, and variables with P < 0.05 were considered statistically significant and retained. Third, correlation analysis was conducted to assess collinearity, and redundant variables were removed based on correlation coefficients and clinical relevance. Variables that satisfied both statistical significance and low collinearity were retained, resulting in a final set of 21 candidate predictors. These variables were subsequently entered into the LASSO regression model.

### Model development strategy

We developed and validated 10 different ML-based models, including:

- K-nearest Neighbors (KNN)
- Logistic Regression (LR)
- Random Forest (RF)
- Decision Tree (DT)
- Artificial Neural Networks (ANN)
- Support Vector Machine (SVM)
- Gradient Boosting Decision Tree (GBDT)
- Light Gradient Boosting Machine (LightGBM)
- Adaptive Boosting (AdaBoost)
- Extreme Gradient Boosting (XGBoost)

The aim was to identify the occurrence of osteopenia among women over 45. All models were implemented using Python (3.10) with the following libraries: sklearn (1.2.2), XGBoost (1.7.4), and LightGBM (4.0.0). We used GridSearchCV to fine-tune the hyperparameters of each model and selected the model that demonstrated the best performance based on the AUC (Area Under the Curve) metric.

Data were split randomly into training (70%) and validation (30%) cohorts using scikit-learn’s train_test_split, ensuring balanced outcome ratios between cohorts. Feature selection procedures were conducted using the training dataset to avoid information leakage and ensure unbiased model evaluation. The models’ predictive accuracy was evaluated using the AUC, sensitivity, specificity, positive predictive value (PPV), negative predictive value (NPV), accuracy, and F-1 score. Calibration curves were generated to evaluate the agreement between predicted probabilities and observed outcomes, thereby assessing model calibration.

### Model simplification

To improve the interpretability of the model, we employed Shapley Additive Explanations (SHAP) values to illustrate how individual variables influenced the identification of osteopenia. This method was used to rank predictor importance, helping to identify the key features in the final model. A decision curve analysis (DCA) was employed to assess the clinical usefulness of the final model, evaluating its net benefit across a range of decision thresholds. The precision-recall (PR) curve was also used to demonstrate the trade-off between precision and recall under different thresholds. Additionally, learning curves were generated to evaluate the model’s ability to generalize.

### Outcomes (evaluation of low bone density)

A single DXA device (Hologic APEX Software, version 4.6.3, Hologic Inc., Bedford, MA, USA) was used to measure BMD at the lumbar spine, femoral neck, and total hip during the study period. Quality control processes were done periodically, such as daily phantom scanning to check the stability of instruments. The study period did not experience any major machine drift. T-scores were calculated using the manufacturer-provided reference database for Asian populations:

- T-value≥−1: Healthy
- T-value<−1: Osteopenia.

T-score ≥ −1.0 was defined as normal bone mineral density, while T-score < −1.0 was defined as osteopenia, according to World Health Organization (WHO) criteria.

### Network calculator

To facilitate clinical application, the final predictive model was integrated into a Shiny application-based web platform. This platform allows healthcare professionals to input relevant clinical features and obtain a probability estimate for osteopenia in women aged 45 years and older.

### Statistical analysis

Continuous variables were reported as mean (± standard deviation) or median (interquartile range), depending on their distribution. Comparisons between groups were performed using the Student’s t-test or Mann-Whitney U test for continuous variables and the Chi-square test or Fisher’s exact test for categorical variables. A significance threshold of 0.05 was used for all two-sided statistical tests.

## Results

### Baseline clinical characteristics

In this study, 1,108 women aged 45 years or older participated in it, with 500 women having normal BMD and 608 having osteopenia. After excluding variables with more than 30% missing data, a total of 48 variables were retained for subsequent feature selection and modeling. **Table 1**, however, presents a more comprehensive set of baseline variables prior to exclusion to allow full comparison between groups. The women with osteopenia were older and had a lower BMI as compared to the normal group (both P < 0.05). There were also significant differences in the bone turnover markers, with the osteopenia group having more TPINP and β-CTX. Moreover, the parameters of metabolic, liver functioning, lipid profile, and renal functioning were observed to differ between the two groups (all P < 0.05). Detailed baseline characteristics are presented in **Table 1**.

### ML model and model interpretation

#### Removing covariates and feature selection

**Figure 2:**
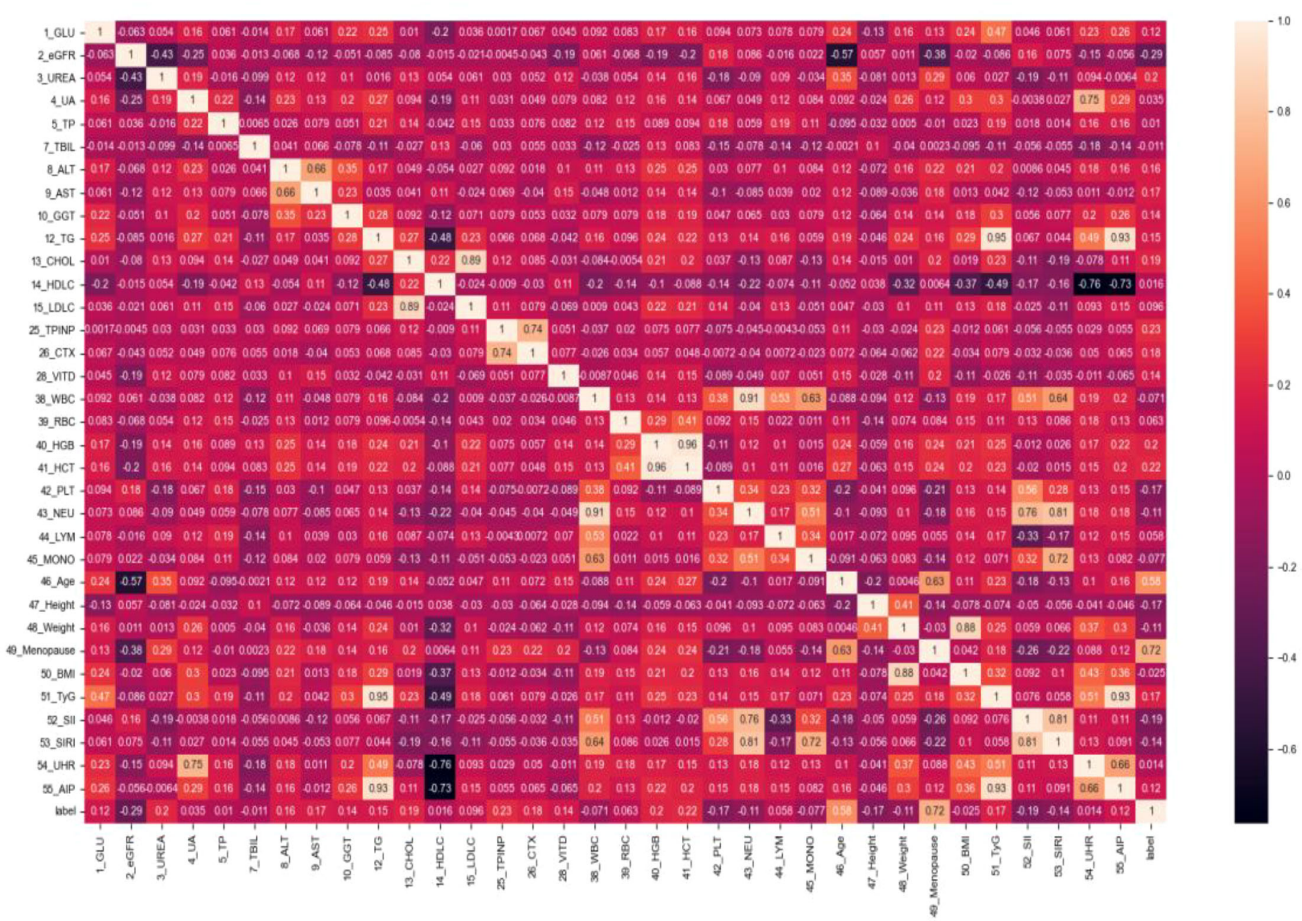
The correlation heatmap between the variables.

The first analysis was an univariate and a multivariate analysis to reveal the variables with a significant association with osteopenia. As **Table 2** shows, a number of variables, such as menopausal status, lipid parameters (CHOL, HDLC, LDLC), bone turnover (β-CTX and VITD), and hematological indices (MONO), were significantly related to osteopenia. Correlation analysis was performed to assess collinearity among continuous variables. As illustrated in **Figure 2**, variables showing moderate-to-high correlation (correlation coefficient > 0.3), in combination with clinical redundancy, were carefully evaluated, and redundant variables were removed. This process resulted in a refined subset of 21 candidate predictors, including age, menopause, BMI, glucose, eGFR, AST, GGT, total cholesterol, HDL-C, LDL-C, PINP, β-CTX, hematocrit, hemoglobin, platelet count, TyG index, SII, SIRI, UHR, AIP, and uric acid. These variables were subsequently used as input for LASSO regression and further feature selection procedures.

**Table 2:** Hazard ratios of selected features for osteopenia.

Feature	Univariate HR	Univariate 95% CI	Univariate P-value	Multivariate HR	Multivariate 95% CI	Multivariate P-value
Age	1.01	1.00-1.02	0.010	0.99	0.98-1.01	0.365
Menopause	20.71	9.82-43.66	<0.001	31.5	13.85-71.63	<0.001
UA (umol/L)	1.00	1.00-1.00	0.001	1.00	1.00-1.01	0.157
TP (g/L)	1.04	1.02-1.06	<0.001	1.05	1.02-1.07	<0.001
CHOL (mmol/L)	1.13	1.05-1.21	0.002	0.51	0.36-0.72	<0.001
HDLC (mmol/L)	1.56	1.22-1.99	<0.001	6.32	1.88-21.27	0.003
LDLC (mmol/L)	1.13	1.04-1.22	0.003	2.21	1.57-3.12	<0.001
TPINP (ng/mL)	1.01	1.00-1.01	<0.001	1.00	0.99-1.00	0.132
β-CTX (ng/mL)	3.04	2.26-4.08	<0.001	2.39	1.55-3.69	<0.001
VITD (ng/mL)	1.02	1.02-1.03	<0.001	1.02	1.01-1.03	<0.001
HGB (g/L)	0.99	0.98-1.00	0.03	0.94	0.91-0.97	<0.001
LYM (×10~9/L)	1.29	1.10-1.52	0.002	1.00	0.51-1.95	0.993
MONO (×10~9/L)	3.97	1.87-8.43	<0.001	23.89	2.44-234.42	0.006

The Cox proportional hazard model was used to calculate HR and 95% CI. Multivariable analysis was done based on significant factors from the univariable analysis.

**Figure 3:**
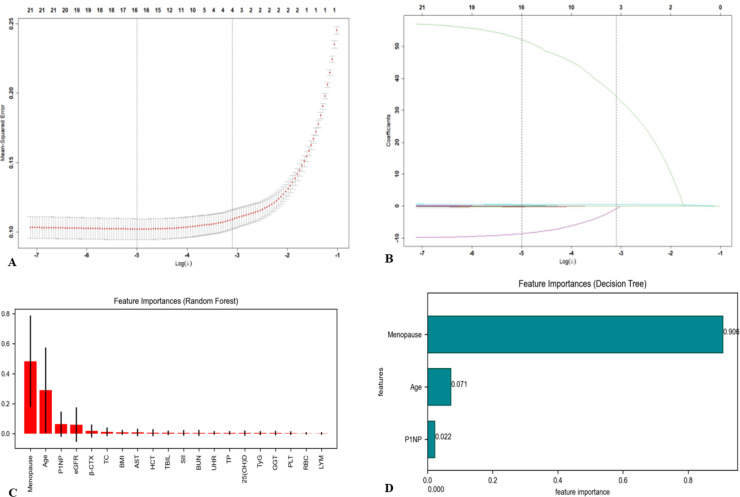
Lasso regression analysis was used to select the characteristic factors. **(A)** A vertical line was drawn at the selected value using 10-fold cross-validation, where the best λ yielded 16 nonzero coefficients. **(B)** In the lasso model, the coefficient contours of 21 texture features are extracted from the log (λ) sequence. Vertical dashed lines are drawn at the minimum mean square error (log (λ) =-4.99647) and the standard error of the minimum distance (log (λ) =-3.09886). **(C)** To compute feature importance for the Random Forest algorithm from the scikit-learn package (in Python). **(D)** The Importance of Attribute Selection Measures in Decision Tree Induction.

LASSO regression analysis was then used to pick the relevant variables further. **Figures 3A, B** were used to determine the 4 main variables (age, menopause, BMI, and PINP) that were selected with the λ 1se criterion that offered a more parsimonious model. Simultaneously, ten significant variables were recognized by using recursive feature elimination (RFE), among them glucose, eGFR, AST, cholesterol, PINP, β-CTX, hematocrit, menopause, TyG, and SII. Even when the number of variables was different, based on the number of variables selected by different methods, the common features between the methods were given priority. Finally, a total of 17 variables were selected for model development using an integrative feature selection strategy. Specifically, variables identified by multiple methods (LASSO, RFE, and random forest importance) were prioritized, and additional variables with established clinical relevance or importance in individual models were retained. Therefore, the final set represents a union of features derived from different selection approaches rather than the output of a single method.

### Feature importance

The red bar chart illustrates the feature importance of the random forest model, highlighting Menopause and Age as the most significant features. Other features, such as TPINP, eGFR, and β-CTX, exhibit relatively lower importance, depicted by red bars of varying heights in the chart. Next to the bar representing the importance of each feature, there is an error line that demonstrates the uncertainty range of the feature importance assessment. In conclusion, the chart primarily underscores the crucial roles of menopause and age in the model, while also offering a reference for the importance of other features, which is equivalent to the conclusion of the decision tree (**Figures 3C, D**).

Variables included in the LASSO regression were preselected based on univariate analysis and clinical relevance, resulting in a reduced set of candidate predictors.

### Evaluation of model performance

A total of 17 candidate variables (eGFR, UREA, AST, GGT, CHOL, TPINP, β-CTX, RBC, HCT, PLT, Age, Height, Menopause, BMI, TyG, SII, UHR) were identified through the combined feature selection process and were initially used for model construction. Although different feature selection methods yielded varying numbers of candidate variables, overlapping features across methods were prioritized, and the final model included a subset of key predictors. Among the ten algorithms with validation cohorts, the top three algorithms in terms of sensitivity were LR (98.9%), AdaBoost (96.7%), and XGboost (95.6%). In terms of specificity, the top three performers were ANN (100%), KNN (86.7%), and DT (82.7%). For accuracy, the top three models were RF (87.4%), GBDT (86.5%), and XGBoost (86.2%). The top three algorithms in terms of F1 score were RF (0.873), GBDT (0.864), and XGBoost (0.859). The RF algorithm was chosen as the best algorithm due to the higher AUC, Accuracy and F1 score than the other models in the validation cohorts (**Table 3**).

**Table 3:** Performance of the ten machine learning algorithms in the training and validation cohorts for osteopenia patients.

Models	AUC	Accuracy	PPV	NPV	Sensitivity	Specificity	F1 score
Training cohort							
KNN	0.772	0.657	0.771	0.587	0.532	0.809	0.652
LR	0.907	0.848	0.837	0.863	0.896	0.789	0.847
RF	0.978	0.929	0.924	0.935	0.948	0.906	0.929
DT	0.931	0.836	0.896	0.779	0.793	0.889	0.836
ANN	0.500	0.451	0.000	0.452	0.000	1.000	0.281
SVM	0.710	0.670	0.705	0.630	0.685	0.651	0.670
GBDT	0.998	0.975	0.979	0.972	0.976	0.974	0.975
LightGBM	0.977	0.916	0.955	0.876	0.889	0.949	0.916
XGboost	1.000	0.999	1.000	0.997	0.998	1.000	0.999
AdaBoost	0.931	0.865	0.831	0.921	0.946	0.766	0.863
Validation cohort							
KNN	0.713	0.550	0.726	0.500	0.290	0.867	0.513
LR	0.869	0.853	0.794	0.981	0.989	0.687	0.848
RF	0.933	0.874	0.851	0.909	0.934	0.800	0.873
DT	0.905	0.802	0.846	0.756	0.781	0.827	0.802
ANN	0.500	0.450	0.000	0.450	0.000	1.000	0.280
SVM	0.722	0.703	0.736	0.665	0.716	0.687	0.703
GBDT	0.929	0.865	0.842	0.901	0.929	0.787	0.864
LightGBM	0.931	0.853	0.853	0.853	0.885	0.813	0.852
XGboost	0.916	0.862	0.821	0.933	0.956	0.747	0.859
AdaBoost	0.910	0.850	0.801	0.946	0.967	0.707	0.846

**Figure 4:**
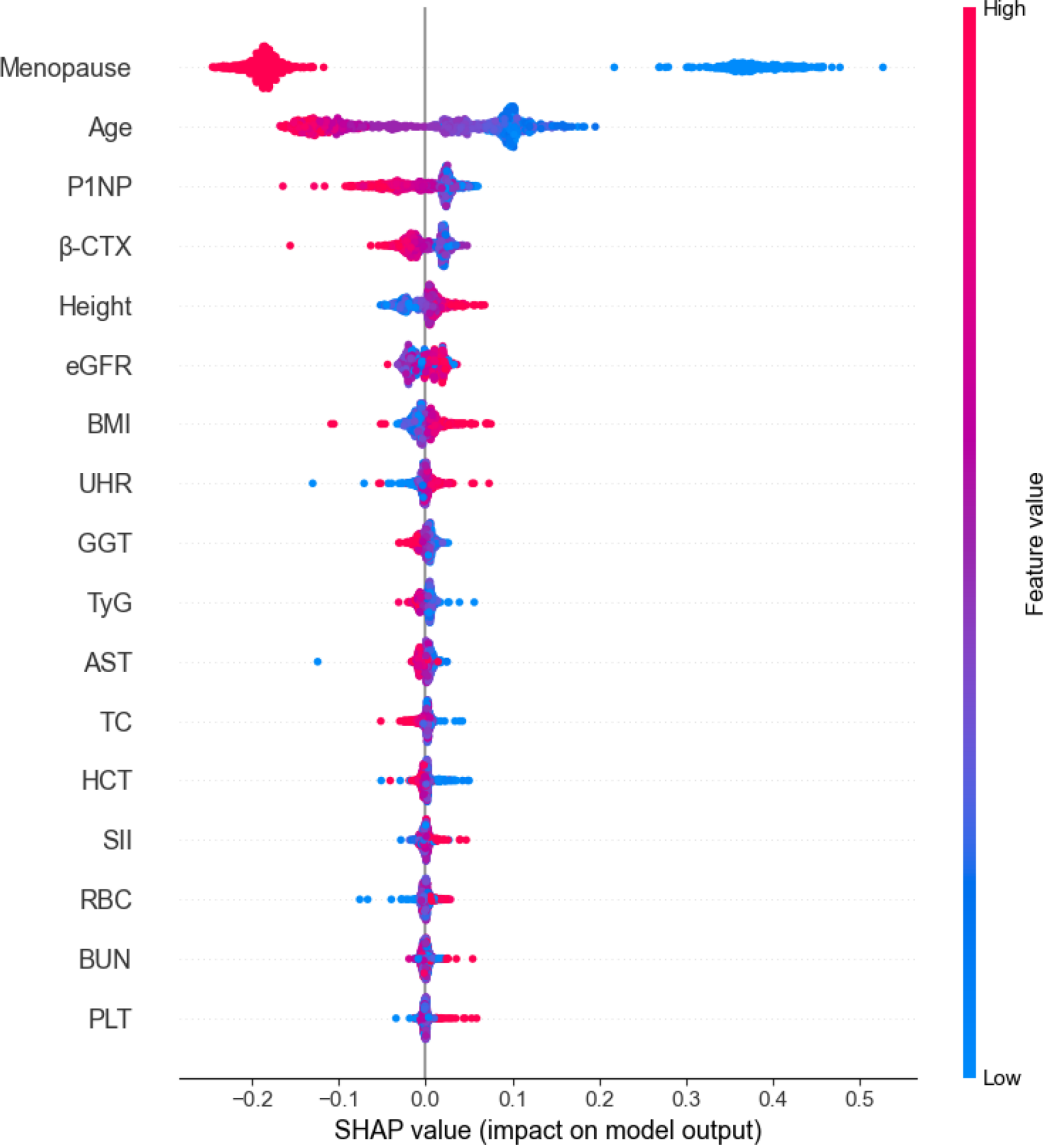
Beeswarm plots of the RF model. Generate SHAP values for each variable and reveal its relationship with osteopenia.

We interpreted the importance of predictor variables based on the SHAP algorithm for the RF model with the best predictive performance. The SHAP value reflects the extent to which a variable contributes to the model. A higher SHAP value of a variable means a higher degree of its contribution to the model. As shown in **Figure 4**, the top-down ordering of the variables means that their contribution to low BMD is in ascending order, with the line with a SHAP value of 0 as the vertical axis. The variables with red color on the right side of the line represent the positive contribution of the variable to the predicted outcome. In contrast, the variables with blue color on the right side of the line have a negative contribution. Therefore, the top six variables in terms of importance for identifying osteopenia in the population were: Menopause > Age > PINP > β-CTX > Height > eGFR, in which Menopause, Age, PINP, and β-CTX were positively correlated with the occurrence of osteopenia, i.e., the older the age, the higher the indexes of PINP and β-CTX, and the higher the probability of developing osteopenia. Height and eGFR were negatively correlated with the occurrence of low BMD, i.e., the lower the eGFR and height, the higher the probability of low BMD. After all, the “menopausal state” is suggested to have a considerable impact on the direction and magnitude of the model’s predictions.

### Model simplification and application

To simplify the prediction models, we advocated the model with the top 6 (Menopause, Age, PINP, β-CTX, Height, eGFR) features, as determined by the SHAP value. To simplify the prediction models, we evaluated the performance of models featuring the top 6 risk factors with the ten algorithms. The results suggested that the model featuring the top 5 (RF, DT, GDBT, LightGBM, AdaBoost) algorithms exhibited a superior performance. The AUC, Accuracy, PPV, NPV, sensitivity, specificity, and F1-score of these models are indicated in **Table 4**.

**Table 4:** Performance comparison of ten algorithms with the top 6 features in the training and validation cohorts for osteopenia patients.

Models	AUC	Accuracy	PPV	NPV	Sensitivity	Specificity	F1 score
Training cohort							
KNN	0.797	0.665	0.824	0.587	0.494	0.871	0.655
LR	0.880	0.854	0.800	0.965	0.979	0.703	0.850
RF	0.929	0.866	0.861	0.873	0.901	0.823	0.865
DT	0.910	0.792	0.888	0.717	0.711	0.891	0.792
ANN	0.867	0.826	0.796	0.877	0.918	0.714	0.823
SVM	0.704	0.666	0.713	0.618	0.654	0.680	0.666
GBDT	0.985	0.943	0.959	0.925	0.936	0.951	0.943
LightGBM	0.932	0.865	0.854	0.879	0.908	0.811	0.864
XGboost	1.000	0.999	1.000	0.997	0.998	1.000	0.999
AdaBoost	0.922	0.846	0.842	0.853	0.887	0.797	0.846
Validation cohort							
KNN	0.726	0.622	0.744	0.556	0.475	0.800	0.614
LR	0.875	0.853	0.791	0.990	0.995	0.680	0.848
RF	0.929	0.874	0.828	0.958	0.973	0.753	0.871
DT	0.902	0.775	0.846	0.712	0.721	0.840	0.775
ANN	0.876	0.847	0.830	0.872	0.907	0.773	0.846
SVM	0.710	0.694	0.748	0.641	0.667	0.727	0.694
GBDT	0.912	0.859	0.833	0.899	0.929	0.773	0.857
LightGBM	0.922	0.859	0.830	0.906	0.934	0.767	0.857
XGboost	0.918	0.850	0.838	0.868	0.902	0.787	0.849
AdaBoost	0.917	0.844	0.823	0.877	0.913	0.760	0.842

#### AUC

**Figure 5:**
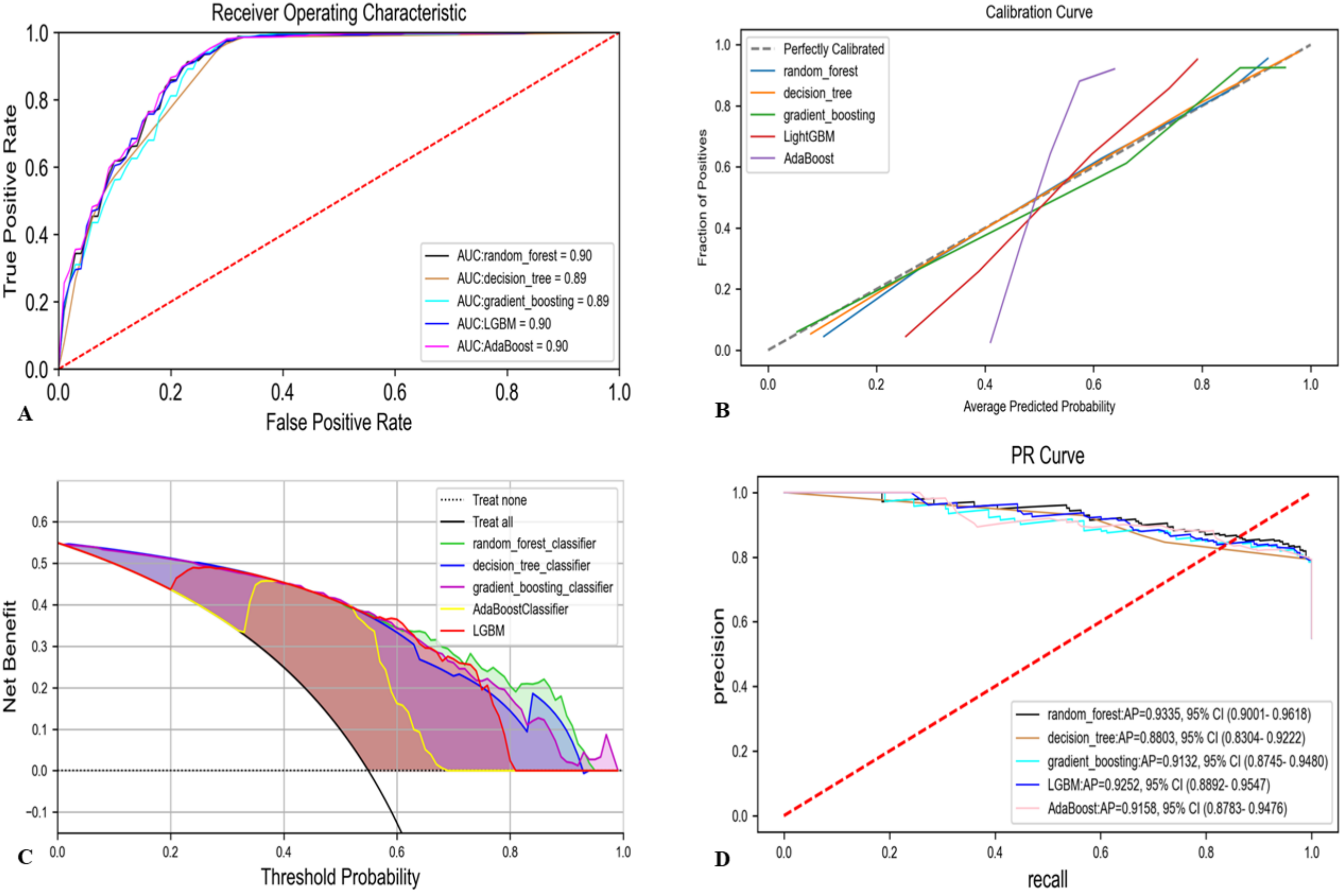
ROC curves **(A)**, calibration curves **(B)**, decision curves **(C)**, PR curve **(D)** for the top five models with six features.

The analysis of ROC curves revealed that no model was lower than the random classification because the curve was above the diagonal (**Figure 5A**). Random forest (RF), LightGBM, and AdaBoost models were the most discriminative, with each having an AUC of around 0.90, compared to decision tree and gradient boosting models, which had lower performance (AUC of around 0.89). All in all, the RF model displayed high and consistent performance, which justifies it as the best model.

#### CC

To test the consistency between predicted probabilities and the actual results, the calibration curves were applied (**Figure 5B**). The random forest (RF) model was the only one that showed a fair performance in terms of calibration, with the predictions being very close to the reference line. Conversely, LightGBM and AdaBoost had a slightly inferior calibration, and decision tree and gradient boosting models had a high degree of deviation, especially in the high probability scales. All in all, the RF model demonstrated higher consistency of prediction in comparison to other models.

#### DCA

The analysis of the decision curve was carried out to determine the clinical usefulness of various models (**Figure 5C**). The RF and gradient boosting models were found to have the greatest net benefit over a variety of threshold probabilities, which shows enhanced clinical utility. The performance of the decision tree model was also acceptable, but a bit less. Even though AdaBoost and LightGBM were not as favorable, they still offered more value compared to the strategies of treating all or treating none. These results imply that the RF model has the most desirable clinical benefit-to-risk ratio.

#### PR curve

The precision–recall (PR) curves were used to evaluate model performance (**Figure 5D**) further. The RF model had the best average precision (AP = 0.9335) and performed better than the other models in most recall ranges. Also, the RF model had a comparatively tight confidence interval, which implies steady performance. LightGBM was ranked​ second, while AdaBoost and gradient boosting exhibited slightly inferior performance. The decision tree model had the weakest performance among the evaluated models.

#### Learning curve

The learning curve constitutes a fundamental tool in the field of ML, utilized to evaluate the training phase of a model. It facilitates the intuitive diagnosis of a model’s fitting and generalization abilities by illustrating the performance trends of the training and validation datasets in correlation with the sample size.

**Figure 6:**
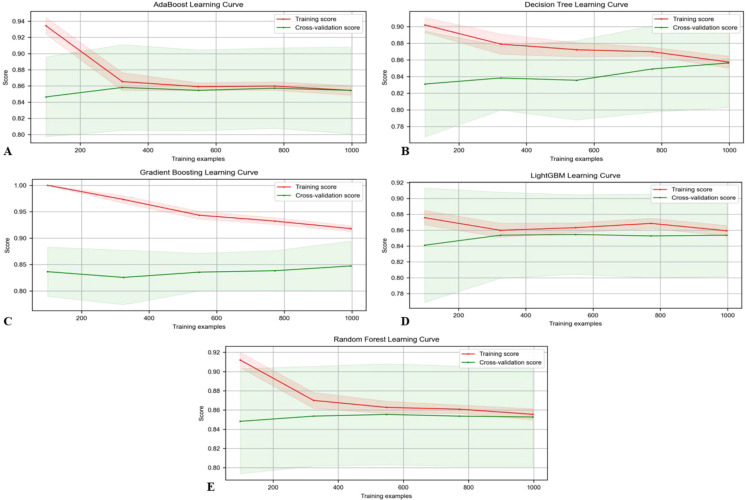
**A–E** Learning curve with the top five algorithms. The red line represents the training set, and the green line represents the validation set. The values are expressed in terms of average and 95% CI. The horizontal axis denotes the quantity of training samples, while the vertical axis represents the score (accuracy). The red line depicts the score trend of the training set, and the green line illustrates the score trend of the 5-fold cross-validation set.

Figure analysis reveals that, apart from the overfitting tendencies observed in decision trees and Gradient Boosting Decision Trees (GDBT), the performance of Adaboost, Light Gradient Boosting Machine (LGBM), and random forest models, as indicated by the red and green lines, converges around an accuracy of 86% with increasing sample size (**Figures 6A-E**). The accuracy is neither notably low nor excessively high, and the substantial overlap between the lines suggests that these three models do not exhibit pronounced underfitting or overfitting. Notably, Adaboost and LGBM models experienced slight fluctuations in their later stages; however, the random forest model demonstrated superior performance compared to the other models.

#### Web-based calculator

**Figure 7:**
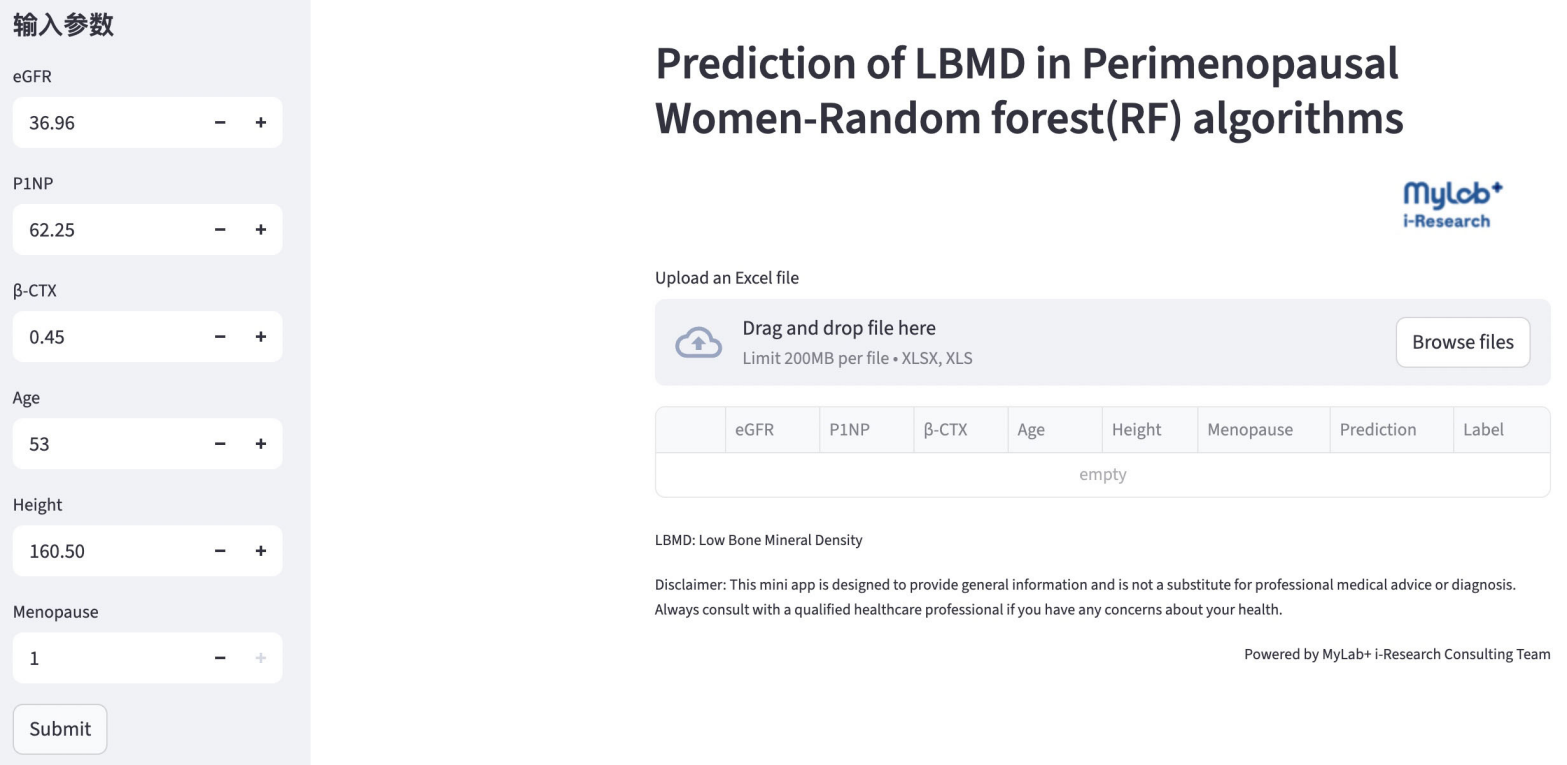
Application screenshot of webpage app.

Based on these findings, we created a web-based calculator (https://55h9qw5jx2qxyo6jjnhg99.streamlit.app/), enabling other researchers to replicate our analyses and validate the model’s performance. A screenshot of the generalized model is provided in **Figure 7**. The comprehensive results indicated that the ML model based on the RF algorithm performed optimally. We have established a simple, effective, and applicable algorithm to assist clinicians in identifying high-risk patients.

## Discussion

As global populations continue to age and lifestyles evolve, osteoporosis has emerged as a critical public health concern, with significant implications for healthcare systems worldwide. The prevalence of osteoporosis is rising rapidly, placing an increasing financial burden on healthcare infrastructure and leading to substantial challenges in patient care (10–12). Despite these growing concerns, osteoporosis remains underdiagnosed and undertreated in several populations, particularly in regions with limited disease awareness and diagnostic resources (13). While this study was conducted in China, its findings may provide preliminary insights for osteoporosis risk assessment in similar populations.

This study proposed an ML-based model designed to identify osteopenia risk in perimenopausal women. By using clinical data from local health institutions, this model addressed the limitations of publicly available databases and concentrated on a region-specific cohort. The model incorporated 17 key predictors, which were selected through rigorous data imputation and feature selection techniques, to forecast osteopenia risk. Key predictors include menopausal status, age, bone turnover markers (PINP and β-CTX), height, and renal function (eGFR), which collectively form the foundation of the predictive algorithm.

Osteoporosis is particularly prevalent among women during the perimenopausal period (14), when a sharp decline in estrogen accelerates bone resorption (15). The process of bone remodeling also becomes less efficient with aging, further exacerbating bone loss. This study highlighted several well-established risk factors, such as age, menopausal status, and BMI, aligning with findings from existing literature (16–18). However, the categorization of menopausal status was based on clinical history rather than hormonal confirmation, which may introduce some degree of misclassification. Notably, the inclusion of biomarkers, such as PINP and β-CTX, represents a novel approach to enhance prediction accuracy. These biomarkers, recognized by the International Osteoporosis Foundation and the International Federation of Clinical Chemistry and Laboratory Medicine as essential for osteoporosis research (19), remain underexplored in clinical prediction models. This study is among the first to incorporate these markers into a risk prediction algorithm specifically for perimenopausal women, providing additional evidence on the potential role of bone turnover markers in identifying low bone mineral density.

The performance of the model was validated using multiple evaluation metrics, including accuracy, sensitivity, specificity, and the AUC. The AUC for our Random Forest (RF) model was 0.933, which compares favorably to similar studies in the field. However, there is a limited body of literature addressing the risk assessment of conventional bone turnover markers (BTMs) in the context of osteopenia. Recent studies have consistently shown that elevated concentrations of bone resorption markers correlate with reduced osteopenia in elderly populations (19, 20), suggesting that maintaining low levels of these markers may be beneficial for skeletal health. Monitoring both BMD and bone turnover markers is crucial for effective osteoporosis management, as changes in bone turnover markers often precede detectable alterations in BMD (21, 22).

In our predictive model, procollagen type I N-terminal propeptide (PINP) and β-crosslaps (β-CTX), which are recommended by the International Osteoporosis Foundation (IOF) and the International Federation of Clinical Chemistry and Laboratory Medicine (IFCC) as reference analytes for bone turnover markers in clinical research (19, 22), emerged as the most significant predictors, ranking among the top three and four variables, respectively, according to Shapley Additive exPlanations (SHAP) analysis. Given these findings, and aided by advanced algorithmic tools, it is feasible to assess the utility of incorporating BTMs into predictive algorithms for assessing the risk of osteopenia. In this context, few studies have examined the use of PINP and β-CTX to reveal low bone mass in perimenopausal women. Since this study is of a cross-sectional nature, the model can be viewed as the classification tool on the present condition of osteopenia and not an indicator of the future risk. The analysis of SHAP even indicated that PINP and β-CTX played an independent role in the prediction model, in addition to the clinical variables (age, menopausal status, height, and eGFR). Although age and menopausal status had the largest overall effect, high PINP and β-CTX levels showed a higher likelihood of osteopenia, which demonstrates the complementary nature of the bone turnover markers in enhancing model performance.

The SHAP analysis further identified BMI as the seventh most influential factor. Data from both the normal and osteopenia groups consistently suggest that lower BMI is associated with increased risk of low BMD across different populations (23, 24). However, the relationship between BMI and BMD is complex, as excessively high BMI may also have detrimental effects (25, 26). Notably, height emerges as a more crucial factor than BMI in the current models and clinical assessments, highlighting its potential to improve the accuracy of bone health evaluations in perimenopausal women. Attention should be given to the bone health of individuals with low BMI, particularly those with reduced stature. Nevertheless, further research is required to validate these findings.

This study aimed to integrate a broad spectrum of clinical indicators into the analysis. As observed in the basic data table, composite indicators, such as renal function (e.g., estimated glomerular filtration rate, eGFR), glucose metabolism (e.g., TyG), inflammatory markers (e.g., systemic immune-inflammation index, SII, and systemic inflammation response index, SIRI), and dyslipidemia (e.g., atherogenic index of plasma, AIP), exhibit significant differences (P<0.01) between the normal and osteopenia groups, as reported in other studies (27–32). After multivariate analysis, eGFR was included in the final model, demonstrating that renal function markers may contribute to the early identification of abnormal BMD in perimenopausal women, potentially enabling timely preventive interventions.

The use of ML-based models for identifying osteoporosis risk has grown significantly. BMD, a key indicator for osteoporosis and osteopenia, is critical in assessing fracture risk, particularly in aging populations. ML techniques have proven effective in predicting, classifying, and managing low BMD, offering methodologies that complement or even surpass traditional diagnostic tools such as Dual-energy X-ray Absorptiometry (DXA). Models, such as logistic regression, gradient boosting, and support vector machines, have shown promise in classifying low BMD using various data sources, including blood biomarkers, anthropometric measurements, and health checkup data. For example, Xu et al. developed a logistic regression model using six variables, including age, BMI, gender, creatine phosphokinase, total cholesterol, and alkaline phosphatase, achieving an area under the receiver operating characteristic curve (AUROC) of 0.785 in a cohort of 3,545 patients. However, the study, which used U.S. National Health and Nutrition Examination Survey (NHANES) data from 2017-2020, did not address age, sex, and racial imbalances (33). Kang et al. developed a gradient boosting model for predicting lumbar BMD in a cohort of 2,026 post-menopausal women and men aged 50 and older from South Korea. Their model, incorporating variables such as body weight, age, alkaline phosphatase (ALP), and osteocalcin, achieved an AUROC of 0.744 (34). In identifying osteopenia in perimenopausal women aged 40 to 55, a neural network model reached an impressive classification accuracy of 92.12% by using 34 selected features, including bioelectrical impedance analysis data (35). However, this model was constrained by the limited sample size (138 clinical datasets) and the large number of features included. Gradient boosting, in particular, has shown superior performance in predicting BMD from genomic data and in elderly male populations (36). In addition, researchers are exploring deep learning frameworks, such as convolutional neural networks (CNNs), to estimate BMD from CT and X-ray images, offering the potential to reduce radiation exposure and enhance diagnostic accuracy (37–41).

The present study also provides an opportunity to compare the performance of various ML algorithms, ranging from traditional regression models to more advanced techniques like Gradient Boosting. While Gradient Boosting has demonstrated superior performance in some studies, our Random Forest model exhibits competitive efficacy in identifying osteopenia, potentially offering a more accessible and clinically feasible option. These findings contribute to the growing body of evidence supporting the use of ML in osteoporosis risk prediction, emphasizing a more nuanced and personalized approach to risk stratification that may complement existing assessment methods such as DXA.

The clinical implications of this study are substantial. For healthcare providers, identifying key predictors of osteopenia, such as menopausal status, age, bone turnover markers, and renal function, enables more precise early interventions for at-risk populations. The web-based tool developed as part of this study allows clinicians to input patient data and receive personalized risk assessments. This feature supports timely management decisions, such as initiating lifestyle changes or increasing the frequency of bone health monitoring.

This model is especially relevant in settings where access to DXA scans is limited. The predictive tool can serve as a cost-effective alternative to imaging, potentially reducing unnecessary referrals for DXA testing and optimizing resource allocation. Furthermore, integrating this model into clinical practice could facilitate early diagnosis of osteoporosis and enable more targeted, preventative treatments, thereby reducing the incidence of osteoporotic fractures and the associated healthcare costs.

On a broader scale, the predictive model could be incorporated into public health campaigns aimed at increasing osteoporosis awareness, particularly in populations with low levels of knowledge about bone health. Educational initiatives directed at both healthcare providers and the general public could highlight the importance of early osteoporosis screening, with a particular focus on perimenopausal women. Community outreach programs could also identify individuals at higher risk, enabling early interventions to prevent bone loss.

The economic implications of this predictive model are significant. In resource-limited regions, where DXA availability is constrained, this model may have potential as a supportive tool for risk stratification, pending further validation. It would help identify individuals at risk of osteopenia and prioritize them for further diagnostic evaluation. This approach could substantially reduce healthcare costs by minimizing unnecessary imaging procedures and focusing resources on high-risk individuals. A detailed cost-effectiveness analysis, especially in resource-limited settings, would be an important next step in assessing the broader applicability of the model.

This study has some limitations. First, as a cross-sectional study, it cannot assess longitudinal changes in bone mineral density or the rate of bone loss over time. Therefore, the identified associations should not be interpreted as causal relationships. Future prospective studies are needed to validate the predictive performance of the model in tracking bone loss progression. Additionally, some clinically relevant variables, including BMD values at different skeletal sites, time since menopause, sex hormone levels (e.g., FSH and estradiol), and lifestyle factors such as smoking and physical activity, were not available in our dataset, which may limit the comprehensiveness of the analysis. Moreover, the current model concentrated exclusively on women without underlying medical conditions; thus, further research is needed to assess its applicability to other populations, including men and individuals with comorbidities, who may present with different risk profiles. Longitudinal studies will also be beneficial to assess the long-term predictive accuracy of the model, particularly in forecasting future fractures or other osteoporosis-related outcomes. This may help further establish the clinical utility of the model in guiding osteoporosis prevention strategies and improving patient outcomes. Moreover, exploring the implementation of the web-based tool in real-world clinical settings may provide valuable insights into its integration into routine practice, especially in primary care environments.

Though the suggested model might prove to be effective in the assessment of risks at the population level, they should exercise caution when using it in the assessment of an individual patient or in longitudinal bone mineral density monitoring since it needs additional validation in prospective and clinical trials. Though a rather great number of candidate variables were initially taken into consideration, feature selection techniques were employed to simplify a model. Nevertheless, it cannot be ruled out that the probability of overfitting exists, and external validation is necessary. Since this study was single-center and was not externally validated, the validity and utility of the model in clinical settings is yet to be determined.

## Conclusion

In conclusion, this study developed a machine learning–based model for identifying osteopenia in women aged 45 years and older using clinical and biochemical data. The random forest model demonstrated good performance in distinguishing individuals with low bone mineral density. The inclusion of bone turnover markers, such as PINP and β-CTX, may provide additional value in osteopenia identification. However, given the single-center design and lack of external validation, further studies are required to confirm the generalizability and clinical applicability of the model.

## Data Availability

The raw data supporting the conclusions of this article will be made available by the authors, without undue reservation.
