# Supplementary material for: An explainable predictive machine learning model of osteopenia for perimenopausal women based on clinical data: a retrospective single-center study
Source: Front Endocrinol (Lausanne). 2026 May 29;17:1817729. doi: 10.3389/fendo.2026.1817729 (PMC13259727; doi:10.3389/fendo.2026.1817729)
Supplement: Supplementary file 1 [file Table1.docx]

**Supplementary Table S1. Percentage of Missing Data for Each Clinical and Laboratory Variable**

| Variable | Missing (%) |
| --- | --- |
| Age | 0 |
| Height | 0 |
| Weight | 0 |
| BMI | 0 |
| Menopausal status | 0 |
| eGFR | 1.2 |
| P1NP | 3.5 |
| β-CTX | 3.8 |
| ALT | 0.9 |
| AST | 1 |
| ALP | 1.5 |
| Calcium | 0.8 |
| Phosphorus | 1.1 |
| Uric acid | 0.7 |
| Glucose | 0.6 |
| Total cholesterol | 0.5 |
| Triglycerides | 0.6 |
| HDL-C | 0.7 |
| LDL-C | 0.8 |
| Hemoglobin | 0.9 |
